# Effectiveness of face-to-face and web-based exercise programs on health outcomes in early postmenopausal women: a randomized trial

**DOI:** 10.1186/s12905-026-04457-4

**Published:** 2026-04-25

**Authors:** Doğukan Kurç, Ayşe Nur Tunalı Van Den Berg

**Affiliations:** Department of Physiotherapy and Rehabilitation, Faculty of Health Sciences, Istanbul Medipol University, Istanbul, Beykoz 34810 Turkey

**Keywords:** Postmenopause, Exercise therapy, Telerehabilitation, Body composition, Depression, Sleep, Quality of life

## Abstract

**Background:**

Oestrogen decline during menopause triggers vasomotor symptoms, weight gain, and mood disturbances. Combined aerobic and resistance exercise can alleviate these symptoms, yet many postmenopausal women face barriers to attending supervised sessions. We compared an eight-week progressive programme of combined aerobic and resistance training delivered face-to-face or via a mobile app against an education-only control in early postmenopausal women.

**Methods:**

Sixty-six early postmenopausal women (aged 45–60) were randomised into face-to-face supervised exercise (*n* = 22), mobile app-based exercise (*n* = 22), or education-only control (*n* = 22). Both exercise groups followed an identical eight-week home-based protocol combining aerobic walking, resistance, core stabilisation, and balance exercises three days per week. Outcomes included menopausal symptoms (MRS), quality of life (SF-36), depressive symptoms (BDI), fatigue (FSS), sleep quality (PSQI), body composition by bioelectrical impedance, and predicted VO₂max. Effect sizes were calculated as Cohen’s d.

**Results:**

Both exercise groups showed significantly greater MRS reductions than controls (face-to-face d = − 1.16; app-based d = − 0.76). Between-group differences in body fat were negligible; muscle mass increased modestly in both exercise groups (d = 0.37–0.38). Predicted VO₂max improved in both groups (d = 1.04–1.08); the web-based group reached significance after Bonferroni correction (*p* = 0.007), while the face-to-face group did not. Fatigue (d = − 1.04 to − 1.66) and sleep quality (d = − 1.08 to − 1.19) improved significantly in both exercise groups. Web-based exercise significantly reduced depressive symptoms (d = − 0.92, *p* = 0.002); face-to-face delivery produced a medium effect (d = − 0.67) that did not reach significance after Bonferroni correction. Differences between delivery methods were negligible to small.

**Conclusions:**

Eight weeks of combined training reduced menopausal symptoms, improved quality of life, psychological well-being, and modestly increased muscle mass and predicted VO₂max. App-based delivery produced comparable effects to face-to-face supervision, though the absence of adherence data limits this comparison. App-based exercise may offer a practical alternative for women unable to attend in-person sessions.

**Trial registration:**

ClinicalTrials.gov, NCT06868134. Registered 20,250,305.

## Background

Menopause, defined as 12 consecutive months of amenorrhea without pathological cause, occurs in most women between 45 and 55 years, with a median age around 51 [1, 2]. The timing of natural menopause varies and is influenced by reproductive and lifestyle factors [3]. It is a natural biological transition, not a disease; nevertheless, the physiological and psychological changes that accompany declining oestrogen levels can significantly affect quality of life and may benefit from targeted lifestyle interventions including exercise, dietary modification, and, where appropriate, hormone replacement therapy. As ovarian oestrogen production declines, changes emerge across several organ systems. Hot flashes and night sweats occur in roughly 80% of women during the menopausal transition; some women experience them for a year or two, while others report symptoms persisting over a decade [4, 5]. Beyond vasomotor complaints, hormonal shifts alter fat distribution, cardiovascular function, and mood [6, 7].

The Study of Women’s Health Across the Nation (SWAN), along with several other landmark longitudinal and cross-sectional studies, has substantially advanced our understanding of these changes [4, 5, 8]. The Massachusetts Women’s Health Study provided early epidemiological evidence linking the menopausal transition to changes in health status and healthcare utilisation [9]. The MONET study characterised changes in energy expenditure and body composition during the transition [10], while the STRIDE longitudinal study documented the impact of menopausal symptoms on health-related quality of life over time [11]. More recently, the FLAMENCO project identified associations between menopausal status and both symptom burden and physical fitness in Southern European women [12], and cross-sectional work by Gould et al. [13] and Moore et al. [14] has characterised differences in exercise metabolism and symptom profiles across pre-, peri-, and postmenopausal women. Baker et al. [15] provided two-year follow-up data on physiological changes spanning the transition, Abildgaard et al. [16] demonstrated reduced whole-body fat oxidation during exercise in postmenopausal women, and Wang et al. [17] provided cross-sectional and longitudinal metabolic profiling evidence characterising the metabolic changes associated with the menopausal transition.

One of the more consistent postmenopausal changes is abdominal fat accumulation, particularly visceral adipose tissue, which raises metabolic concern because visceral fat correlates with metabolic syndrome [18, 19]. Cardiovascular risk rises as well, since oestrogen previously supported healthier lipid profiles, better endothelial function, and lower systemic inflammation [20]. Although circulating oestrogen levels stabilise at low concentrations in early postmenopause, the cumulative physiological consequences of oestrogen withdrawal — including redistribution of adipose tissue, accelerated bone resorption, and altered lipid metabolism — continue to unfold during the first five years after the final menstrual period [13, 15]. This makes early postmenopause a critical intervention window, as these changes are still actively progressing and potentially modifiable through exercise. Depression and anxiety tend to increase during perimenopause and early postmenopause, with hormonal fluctuations heightening susceptibility to mood disorders [21, 22]. Nearly half of menopausal women also report sleep problems, which can amplify psychological distress; indeed, depression, fatigue, and poor sleep often cluster during this transition and reinforce one another [23, 24].

Exercise has received growing attention as a non-pharmacological approach to managing menopausal symptoms. Trials and meta-analyses link regular physical activity with fewer vasomotor episodes, better mood, improved sleep, and healthier body composition [25–28]. Several intervention studies have demonstrated the benefits of structured exercise in postmenopausal populations. Aiello et al. [29] reported improvements in body composition following a year-long moderate-intensity exercise programme, while the Cochrane review by Daley et al. [30] examined the evidence for exercise as a treatment for vasomotor symptoms and concluded that physical activity may be beneficial, though the evidence base remained limited. Nguyen et al. [31] systematically reviewed the effects of physical activity on quality of life during menopause, and a recent overview of reviews by Money et al. [32] found some evidence supporting yoga and aerobic exercise for symptom relief but concluded that evidence was insufficient to recommend a specific exercise modality. Yılmaz Babacan et al. [33] recently demonstrated significant reductions in vasomotor symptoms and improvements in quality of life following 12 weeks of combined resistance and aerobic training in postmenopausal women.

Current physical activity guidelines from the American College of Sports Medicine recommend that all adults — including postmenopausal women — perform at least 150 min per week of moderate-intensity aerobic exercise combined with resistance training for major muscle groups two to three times weekly [34, 35]. However, no menopause-specific exercise guidelines currently exist, which may limit clinicians’ ability to provide targeted advice and women’s confidence in engaging with exercise during this transition. Many women cannot attend supervised sessions regularly due to symptom burden, scheduling conflicts, transportation difficulties, and facility costs. The Women in Sport “Menopause, Me, and Physical Activity” report [36] documented that menopausal symptoms themselves — including fatigue, joint pain, and low confidence — serve as significant barriers to exercise participation. McNulty et al. [37] identified similar barriers and facilitators in a qualitative study of perimenopausal women, highlighting the need for flexible, accessible delivery models. The COVID-19 pandemic accelerated interest in remote alternatives, and mobile platforms such as PhysiApp now allow clinicians to prescribe video-guided exercise programmes and track adherence from a distance [38–41].

We conducted a three-arm randomised controlled trial to test the hypothesis that structured exercise delivered via either face-to-face supervision or a mobile application would improve menopausal symptoms and related health outcomes compared with education alone. We hypothesised that both face-to-face supervised and app-based exercise would produce greater improvements in menopausal symptoms (primary outcome), quality of life, psychological well-being, body composition, and predicted VO₂max (secondary outcomes) compared with an education-only control, and that the two exercise delivery methods would yield comparable effects.

## Methods

### Study design and setting

This study was a three-arm, parallel-group, randomised controlled trial conducted at Istanbul Medipol University, Istanbul, Turkey. The trial compared the effectiveness of face-to-face supervised exercise, web-based exercise delivered via PhysiApp (Physitrack Ltd., London, UK), and an education-only control condition in early postmenopausal women.

The study protocol received ethical approval from the Istanbul Medipol University Non-Interventional Clinical Research Ethics Committee (decision no: 643, date: 10 August 2023) and was registered with ClinicalTrials.gov (NCT06868134; registered 2025-03-05). The trial was registered retrospectively; although participant enrolment commenced in September 2023, registration was delayed due to the corresponding author’s unfamiliarity with international registration requirements at the time of study initiation. The study protocol was not modified between the start of enrolment and registration. All procedures adhered to the Declaration of Helsinki, and written informed consent was obtained from each participant prior to enrolment.

The trial design and reporting followed the Consolidated Standards of Reporting Trials (CONSORT) 2025 statement [42]. Participants were recruited between September 2023 and June 2024 through printed and electronic announcements posted at Istanbul Medipol University-affiliated outpatient physiotherapy and gynaecology clinics and at community health centres in the Beykoz and Çekmeköy districts of Istanbul.

An a priori sample size calculation was performed using G*Power software (version 3.1.9.7; Heinrich-Heine-Universität Düsseldorf, Germany). Based on a one-way ANOVA design with three groups, an effect size of f = 0.42 was assumed based on MRS total score reductions reported in a comparable exercise trial in postmenopausal women [33]. With α = 0.05 and statistical power of 80%, a minimum of 60 participants (20 per group) was required.

Eligible participants were randomly allocated to one of three groups in a 1:1:1 ratio using a computer-generated random number sequence. Randomisation was performed by an independent researcher who had no involvement in participant recruitment or outcome assessment. Due to the nature of the intervention, blinding of participants and the treating physiotherapist was not feasible; however, the outcome assessor was blinded to group allocation.

## Participants

**Fig. 1:**
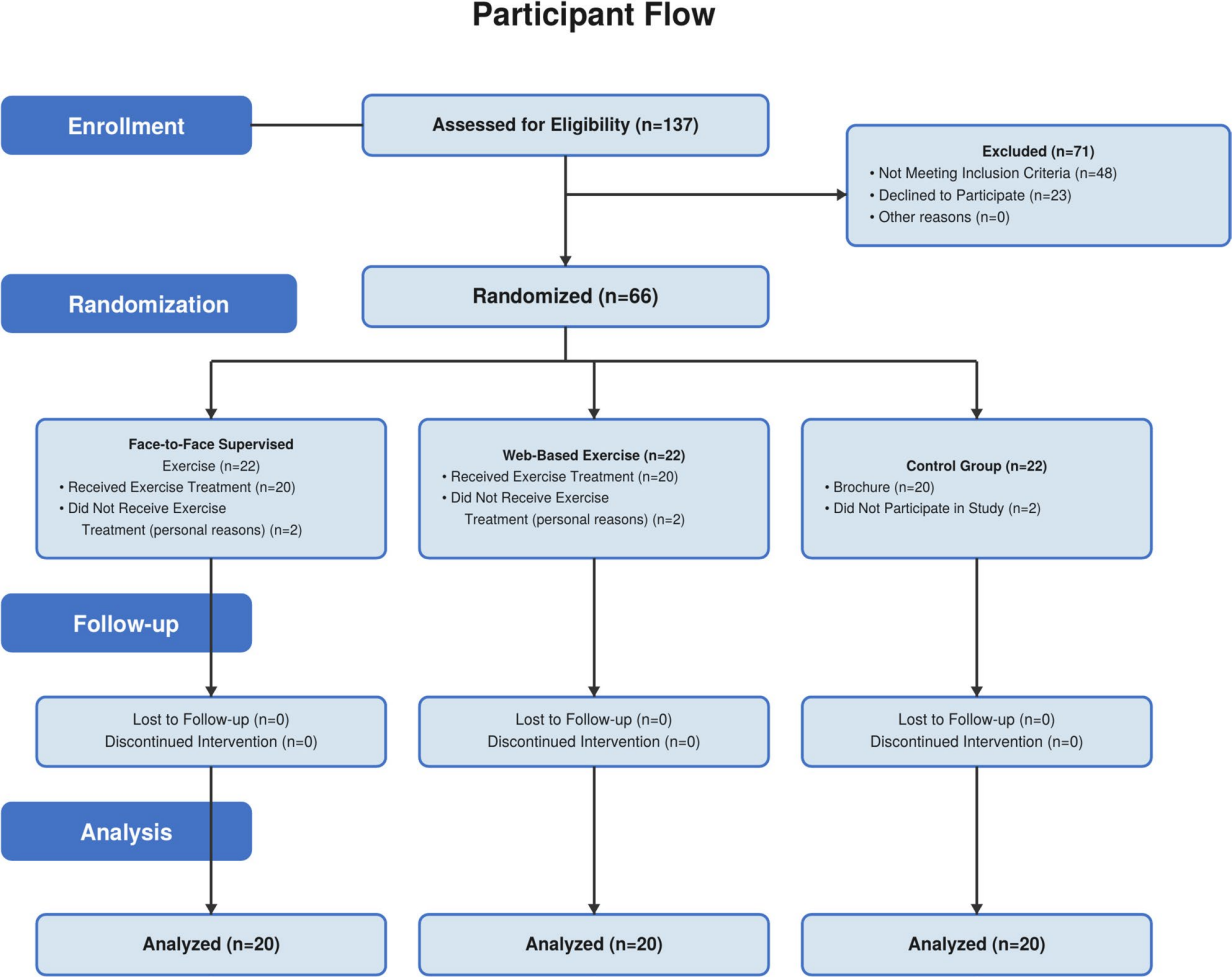
CONSORT flow diagram showing participant recruitment, allocation, follow-up, and analysis

A total of 137 women were screened for eligibility between September 2023 and June 2024 according to the inclusion and exclusion criteria presented in Table 1. Of these, 66 met all criteria and were randomised. Six participants withdrew before receiving any intervention: two in the face-to-face group (one due to a family emergency, one due to scheduling conflicts), two in the web-based group (one relocated, one cited personal reasons), and two in the control group (both declined further participation without specifying a reason). The remaining 60 participants (20 per group) completed the full 8-week protocol with no further attrition. The flow of participants through screening, allocation, and follow-up is presented in the CONSORT diagram (Fig. 1).

**Table 1:** Inclusion and exclusion criteria with rationale

Criterion	Type	Rationale
Aged 45–60 years	Inclusion	Covers the typical early postmenopausal age range [1, 2]
BMI < 40 kg/m²	Inclusion	Excludes morbid obesity that may limit exercise capacity
Natural menopause within preceding 5 years (amenorrhea ≥ 12 months)	Inclusion	Defines early postmenopause as the target intervention window [1]
Ability to use a smartphone/tablet and access mobile applications	Inclusion	Required for web-based group participation
System Usability Scale (SUS) score ≥ 68	Inclusion	Ensures adequate digital literacy for app-based intervention
Willingness to provide written informed consent	Inclusion	Ethical requirement
Severe visual impairment not correctable with standard aids	Exclusion	May impair ability to follow exercise demonstrations
Significant musculoskeletal disorder or severe joint limitation	Exclusion	Contraindication to prescribed exercises
Cognitive impairment identified through clinical evaluation	Exclusion	May impair comprehension of instructions and questionnaires
Serious cardiovascular disease or medical contraindication to exercise	Exclusion	Safety consideration [34]
Current enrolment in another structured exercise programme	Exclusion	Would confound the intervention effect
Any clinical or psychosocial condition interfering with study adherence	Exclusion	Minimises non-compliance-related attrition

### Interventions

All participants were assessed at baseline (T0) and at the end of the 8-week intervention period (T1). All assessments were conducted in the morning (08:00–11:00) at the university research laboratory to minimise diurnal variation. The study timeline, including measurement points, supervised sessions, and exercise delivery for each group, is presented in Fig. 3. The two exercise groups followed identical progressive combined aerobic and resistance training programmes; only the delivery method differed.

#### Control group

Participants received an illustrated educational brochure titled “Menopause: A New Beginning,” developed in accordance with World Health Organization recommendations, covering physiological changes during menopause, benefits of physical activity, and lifestyle modifications. No structured exercise programme or additional supervision was provided.

#### Face-to-face supervised exercise group

Participants received an 8-week progressive home-based exercise programme with in-person supervision. Face-to-face sessions were scheduled at Weeks 0, 2, 4, and 6, during which a physiotherapist demonstrated the exercises, corrected technique errors, and progressed intensity according to the protocol. Between supervised sessions, participants performed the prescribed exercises independently at home. All 20 participants attended all four scheduled supervised sessions.

#### Web-based exercise group

Participants received the same programme delivered through PhysiApp (Physitrack Ltd., London, UK). Each participant received a personal access code to log into the mobile application, which displayed video demonstrations with written instructions and audio cues in Turkish. Automated push notifications were sent on scheduled exercise days.

#### Exercise protocol

Both exercise groups followed an identical 8-week progressive programme combining aerobic and resistance training components, performed 3 days per week on non-consecutive days. The detailed week-by-week programme is provided in Supplementary Table S1.

##### Aerobic component

Brisk walking at moderate intensity (RPE 11–13 on the Borg scale, corresponding to 40–60% of heart rate reserve). Duration: 15 min in Weeks 1–4, progressing to 20–30 min in Weeks 5–8.

##### Resistance and balance component

**Fig. 2:**
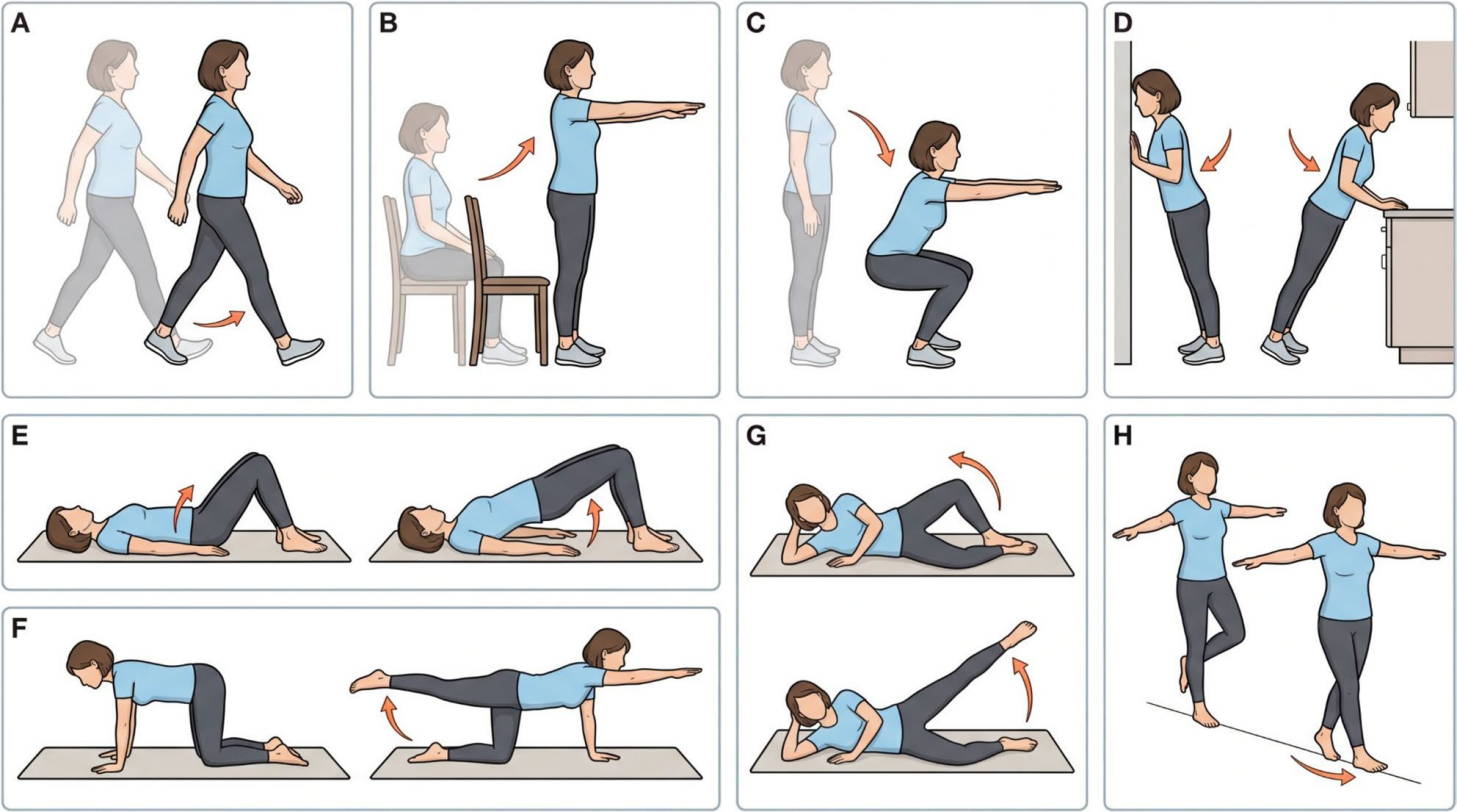
Progressive exercise programme. **A** Brisk walking; (**B**) Sit-to-stand with chair support; (**C**) Bodyweight squat; (**D**) Wall push-up progressing to counter push-up; (**E**) Pelvic tilt progressing to glute bridge; (**F**) Bird-dog from tabletop position; (**G**) Clamshell progressing to side-lying hip abduction; (**H**) Single-leg stance progressing to tandem walk. Panels **B**–**C** and left/right sub-panels in **D**–**H** illustrate the progression from Weeks 1–4 to Weeks 5–8

Exercises targeting major muscle groups (see Fig. 2 for illustrations): chair sit-to-stand progressing to bodyweight squat, wall push-up progressing to counter push-up, pelvic tilt and bridging, bird-dog exercise, clamshell and side-lying hip abduction, and single-leg stance with tandem walking. Initial prescription: 2 sets × 10 repetitions per exercise. Progression at Week 5: 3 sets × 12 repetitions with increased difficulty through technique modifications.

#### Adherence

All face-to-face participants attended all four supervised sessions (100% supervised session attendance). Session-by-session home exercise logs were not collected in either group. For the web-based group, the platform’s data retention policy led to automatic deletion of usage records after the study period, preventing retrospective retrieval of objective digital adherence data.

### Outcome measures

All outcomes were assessed at baseline and at Week 8 by a trained assessor blinded to group allocation. All questionnaires were administered in paper format at the university research laboratory under supervision. All instruments have been validated for paper-based administration in Turkish populations.

#### Primary outcome

Menopause Rating Scale (MRS): An 11-item self-administered questionnaire evaluating menopausal symptom severity across three domains: somatic-vegetative (e.g., hot flushes, cardiac complaints, joint pain), psychological (e.g., depressed mood, irritability, anxiety), and urogenital (e.g., sexual problems, urinary complaints) [43]. Each item is rated on a 5-point Likert scale (0 = absent to 4 = very severe), yielding a total score of 0–44; higher scores indicate greater symptom burden. The MRS demonstrates good internal consistency (Cronbach α = 0.83) and test–retest reliability across diverse populations. Turkish validation was performed by Gürkan [44].

#### Secondary outcomes

Health-related quality of life (SF-36): A 36-item generic health survey assessing eight domains: physical functioning, role limitations due to physical problems, bodily pain, general health perceptions, vitality, social functioning, role limitations due to emotional problems, and mental health [45]. Each domain is scored from 0 to 100, with higher scores indicating better health status. The SF-36 is one of the most widely used instruments in clinical trials for evaluating the multidimensional impact of health interventions. Turkish adaptation and validation were performed by Koçyiğit et al. [46].

##### Depressive symptoms (Beck Depression Inventory — BDI)

A 21-item self-report instrument designed to assess the presence and severity of depressive symptoms over the past two weeks [47]. Each item is scored from 0 to 3, yielding a total score of 0–63, with established cut-off ranges: 0–9 minimal, 10–18 mild, 19–29 moderate, and 30–63 severe depression. The BDI is widely used in clinical and research settings due to its strong psychometric properties and sensitivity to change. Turkish validation was performed by Hisli [48].

##### Fatigue (Fatigue Severity Scale — FSS)

A 9-item self-report instrument assessing the impact of fatigue on daily functioning and motivation over the preceding week [49]. Each item is rated on a 7-point Likert scale (1 = strongly disagree to 7 = strongly agree), and the final score is calculated as the mean of all items; a mean score ≥ 4 is considered clinically significant fatigue. The FSS has demonstrated high internal consistency and has been validated across multiple chronic conditions. Turkish adaptation was performed by Armutlu et al. [50].

##### Sleep quality (Pittsburgh Sleep Quality Index — PSQI)

A 19-item self-report instrument evaluating sleep quality and disturbances over the preceding month across seven component scores: subjective sleep quality, sleep latency, sleep duration, habitual sleep efficiency, sleep disturbances, use of sleeping medication, and daytime dysfunction [51]. Component scores are summed to produce a global score ranging from 0 to 21, where a score > 5 distinguishes poor from good sleepers with high diagnostic sensitivity and specificity. The PSQI is widely recommended for clinical research involving sleep-related outcomes. Turkish validation was performed by Ağargün et al. [52].

##### Body composition

Assessed using a multifrequency segmental bioelectrical impedance analyser (TANITA BC-545 N, Tanita Corporation, Tokyo, Japan), which estimates body composition by measuring resistance to a low-level electrical current passed through hand and foot electrodes. Standardised pre-test conditions were enforced: participants fasted for ≥ 4 h, avoided vigorous exercise for 24 h, and emptied their bladder immediately before measurement, in accordance with manufacturer guidelines. Reported variables included body fat percentage and total muscle mass (kg).

##### Predicted VO₂max

Ebbeling single-stage treadmill walking test [53]. Participants walked at a self-selected pace (2.0–4.5 mph) on a 5% grade for 4 min. Steady state was defined as heart rate variation ≤ 5 bpm during the final two minutes. Heart rate was monitored using a wrist-worn optical heart rate monitor (Xiaomi Mi Band 7) and cross-checked with treadmill grip sensors; wrist-worn values were used for calculations. Maximal exercise testing was not performed because it requires medical supervision with emergency resuscitation equipment not available at the assessment site; submaximal protocols provide acceptable estimates in low-risk populations [53]. The equation.

$$VO_{2}max \left(ml/kg/min\right)=15.1+\left(21.8\times{speed}\right) - \left(0.327\times {HR}\right) -\left(0.263\times speed\times age\right) +\left(0.00504 \times{HR}\times{age}\right) +\left(5.98\times sex\right)$$

where speed is in mph, HR is steady-state heart rate, age in years, and sex = 0 for females.

### Statistical analysis

Analyses were performed using NCSS 2007 (Kaysville, Utah, USA). Continuous variables are presented as mean ± SD; categorical variables as frequency (%). Shapiro-Wilk tests indicated that the majority of outcome variables were not normally distributed (*p* < 0.05); therefore, non-parametric tests were used for all analyses.

Between-group comparisons were conducted using the Kruskal-Wallis test. When significant differences were detected, pairwise post-hoc comparisons were performed using the Mann-Whitney U test with Bonferroni correction (adjusted α = 0.017). The Mann-Whitney U test was selected over Dunn’s test because it provides exact p-values for each pairwise comparison and is widely used in rehabilitation research with similar sample sizes; both approaches are methodologically appropriate following a significant Kruskal-Wallis result. Within-group changes were analysed using the Wilcoxon signed-rank test.

Effect sizes were calculated as Cohen’s d for pairwise between-group comparisons of change scores. Interpretation: negligible (|d| < 0.2), small (0.2 ≤ |d| < 0.5), medium (0.5 ≤ |d| < 0.8), or large (|d| ≥ 0.8) [54]. We report both p-values and Cohen’s d because they serve complementary purposes: p-values indicate statistical significance given the sample size, while effect sizes quantify practical magnitude independent of sample size. In a trial of this size (*n* = 20 per group), p-values may fail to reach significance for genuinely meaningful effects, making effect sizes essential for clinical interpretation.

All analyses were conducted on a modified intention-to-treat basis (*n* = 60). Significance: *p* < 0.05 (two-tailed).

### Use of AI-assisted tools

**Fig. 3:**
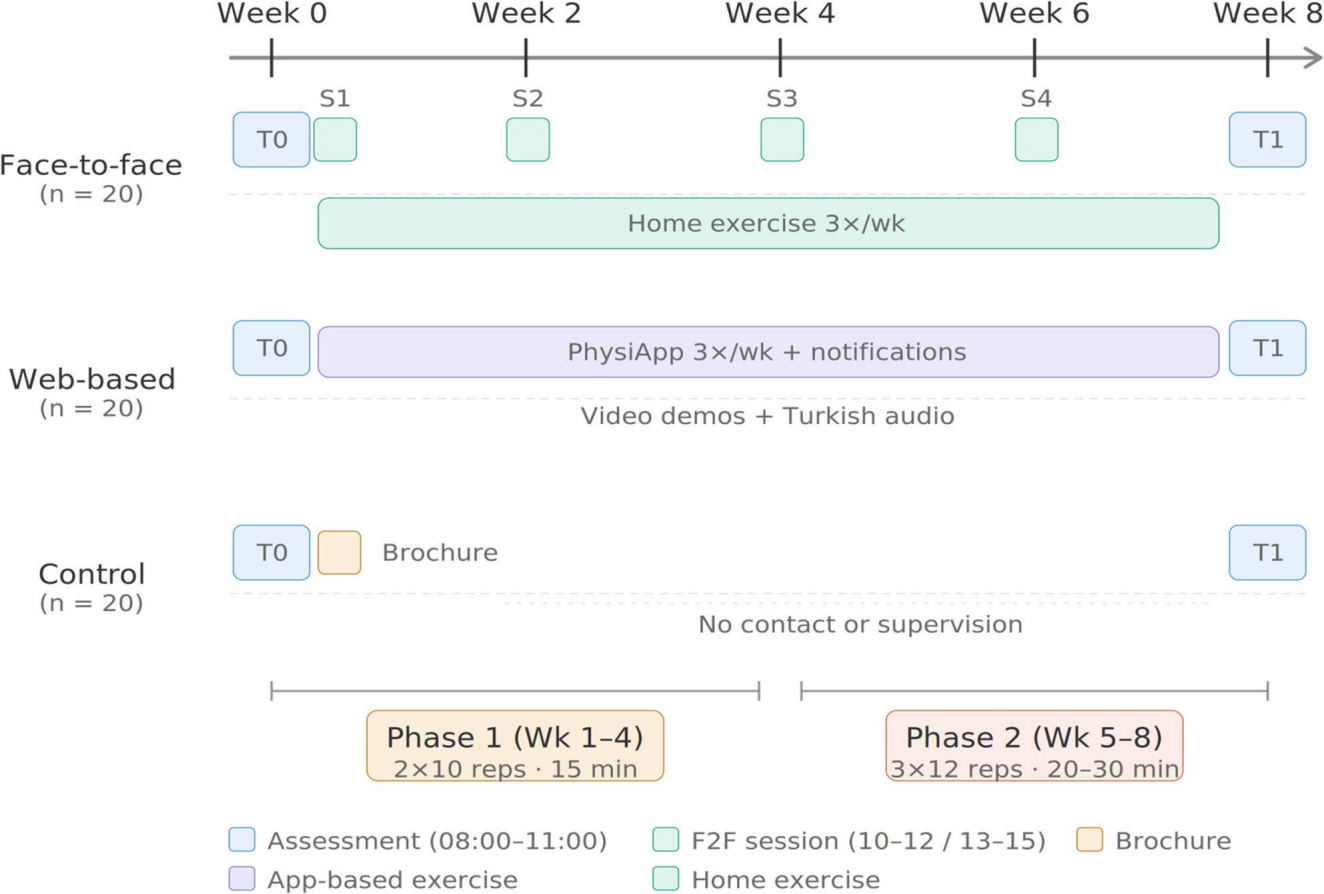
Study timeline schematic showing measurement points (T0, T1), intervention delivery, and exercise progression for each group. S1–S4: supervised physiotherapy sessions at Weeks 0, 2, 4, and 6. All assessments were conducted between 08:00 and 11:00. Supervised sessions were conducted between 10:00–12:00 or 13:00–15:00

The original manuscript was drafted in Turkish and translated into English with the assistance of large language models (ChatGPT, OpenAI; and Claude, Anthropic). The exercise illustrations in Fig. 2 were generated using an AI image generation tool (Gemini, Google DeepMind) based on detailed prompts describing each exercise position, and were reviewed for anatomical accuracy by the authors. The study timeline schematic (Fig. 3) was created with the assistance of Claude (Anthropic). All AI-generated content was reviewed, edited, and verified for accuracy by all authors, who take full responsibility for the content of the manuscript.

## Results

### Participant characteristics

Baseline demographic and anthropometric characteristics are presented in Table 2. No statistically significant differences were observed among the three groups for age (*p* = 0.786), height (*p* = 0.753), body weight (*p* = 0.276), or BMI (*p* = 0.155).

**Table 2:** Baseline demographic and anthropometric characteristics

Variable	Face-to-Face (*n* = 20)	Web-Based (*n* = 20)	Control (*n* = 20)	*p*
Age (years)	55.15 ± 3.33	54.65 ± 3.13	54.15 ± 4.61	0.786
Height (m)	1.60 ± 0.07	1.61 ± 0.05	1.59 ± 0.05	0.753
Body weight (kg)	77.67 ± 8.93	74.70 ± 9.08	73.70 ± 12.09	0.276
BMI (kg/m²)	30.48 ± 3.03	28.89 ± 3.15	28.93 ± 4.19	0.155
Education, n (%)				
Literate only	1 (5%)	1 (5%)	0 (0%)	—
Primary school	6 (30%)	4 (20%)	5 (25%)	—
High school	7 (35%)	6 (30%)	9 (45%)	—
University	5 (25%)	7 (35%)	4 (20%)	—
Postgraduate	1 (5%)	2 (10%)	2 (10%)	—
Marital status, n (%)				
Married	15 (75%)	15 (75%)	14 (70%)	—
Single	2 (10%)	1 (5%)	1 (5%)	—
Widowed/Divorced	3 (15%)	4 (20%)	5 (25%)	—
Employed, n (%)	8 (40%)	7 (35%)	9 (45%)	—
Current or former smoker, n (%)	4 (20%)	5 (25%)	3 (15%)	—

Continuous data as mean ± SD; categorical data as n (%). Between-group comparisons by Kruskal-Wallis (continuous) or descriptive (categorical)

### Primary outcome: menopausal symptoms (MRS)

Both exercise groups demonstrated significantly greater reductions in MRS total scores compared with the education-only control; baseline values, change scores, and between-group comparisons for all MRS subscales are detailed in Table 3.

**Table 3:** Menopause rating scale scores: baseline, change, and between-group comparisons

MRS Subscale	Group	Baseline	Δ (Change)	Within-group *p*	KW-H (Δ)	*p* (Δ)	Post-hoc
Total	F2F	24.05 ± 4.84	−12.90 ± 7.45^a^	**0.001**	11.879	**0.003**	F2F vs. C: *p* = 0.001; Web vs. C: *p* = 0.004
	Web	22.55 ± 4.41	−9.95 ± 6.63^a^	**0.001**			
	Control	20.30 ± 3.25	−5.30 ± 5.51	**0.001**			
Somatic	F2F	9.85 ± 2.03	−5.25 ± 3.09^a^	**0.001**	10.986	**0.004**	F2F vs. C: *p* = 0.002; Web vs. C: *p* = 0.008
	Web	9.05 ± 1.88	−3.95 ± 2.78^a^	**0.001**			
	Control	8.10 ± 1.45	−2.10 ± 2.36	**0.002**			
Psychological	F2F	8.55 ± 1.88	−4.55 ± 2.93^a^	**0.001**	11.248	**0.004**	F2F vs. C: *p* = 0.001; Web vs. C: *p* = 0.006
	Web	7.95 ± 1.64	−3.45 ± 2.52^a^	**0.001**			
	Control	7.10 ± 1.25	−1.75 ± 2.07	**0.002**			
Urogenital	F2F	5.65 ± 1.04	−3.10 ± 1.52^a, b^	**0.001**	11.063	**0.004**	>F2F vs. C: *p* = 0.001; Web vs. C: *p* = 0.005
	Web	5.55 ± 1.00	−2.55 ± 1.47^a^	**0.001**			
	Control	5.10 ± 0.72	−1.45 ± 1.32	**0.001**			

^a^ Significantly greater change than control (*p* < 0.017, Bonferroni-corrected)

^b^ Significantly greater change than web-based group. Δ = post − baseline; more negative = greater improvement

Bold values indicate statistical significance (*p* < 0.05)

**Fig. 4:**
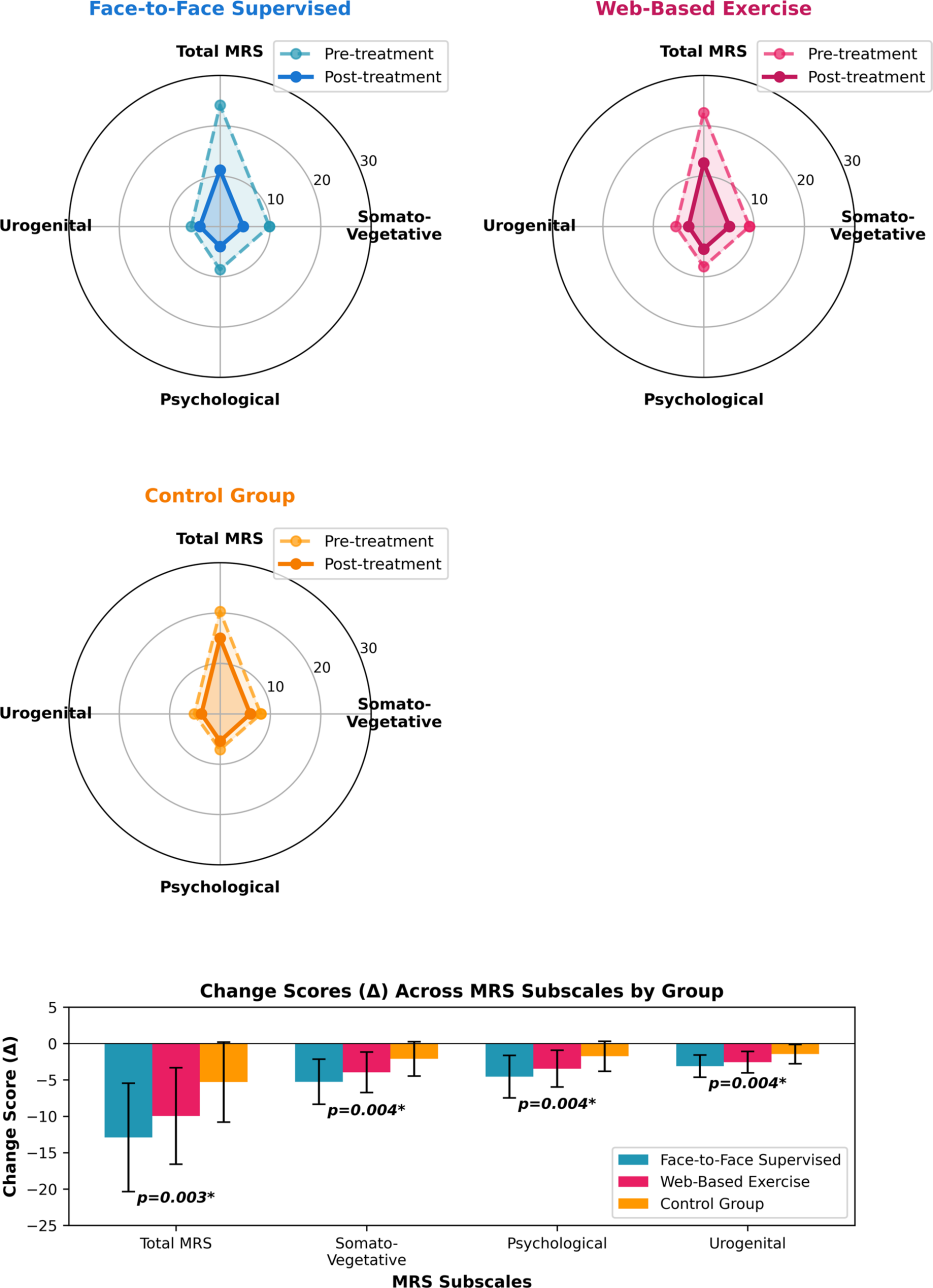
Radar plots showing normalised pre-treatment and post-treatment MRS subscale scores for each group (top panels) and change scores across subscales (bottom panel). More negative values indicate greater symptom reduction

MRS subscale scores and change scores are illustrated in Fig. 4.

### Secondary Outcomes

Depressive symptoms, fatigue severity, and sleep quality showed notable improvements in both exercise groups; baseline values, change scores, and post-hoc comparisons are presented in Table 4.

**Table 4:** Depression, fatigue, and sleep quality: baseline, change, and between-group comparisons

Outcome	Group	Baseline	Δ (Change)	Within-group *p*	KW-H (Δ)	*p* (Δ)	Post-hoc
BDI	F2F	16.00 ± 6.22	−5.65 ± 4.53	**0.002**	10.323	**0.006**	>Web vs. C: *p* = 0.002
	Web	19.00 ± 7.80	−7.30 ± 6.67^a^	**0.001**			
	Control	15.65 ± 6.39	−2.20 ± 4.76	0.066			
FSS	F2F	4.95 ± 1.51	−0.81 ± 0.80^a^	**0.001**	13.386	**0.001**	F2F vs. C: *p* = 0.003; Web vs. C: *p* = 0.001
	Web	5.38 ± 1.27	−0.96 ± 0.60^a^	**0.001**			
	Control	3.65 ± 1.50	−0.21 ± 0.43	**0.013**			
PSQI	F2F	12.05 ± 4.55	−3.15 ± 2.39^a^	**0.001**	19.336	**0.001**	F2F vs. C: *p* = 0.001; Web vs. C: *p* = 0.001
	Web	11.45 ± 4.11	−3.55 ± 2.66^a^	**0.001**			
	Control	9.75 ± 5.20	−0.35 ± 2.39	0.264			

^a^Significantly greater change than control. Lower scores = better for all measures

Bold values indicate statistical significance (*p* < 0.05)

Body composition and cardiorespiratory fitness were assessed as secondary physiological outcomes; baseline values, change scores, and between-group comparisons are presented in Table 5.

**Table 5:** Body composition and Predicted VO₂max: baseline, change, and between-group comparisons

Variable	Group	Baseline	Δ (Change)	Within-group *p*	KW-H (Δ)	*p* (Δ)	Post-hoc
Body fat (%)	F2F	33.91 ± 4.35	−1.72 ± 1.54	**0.001**	3.241	0.198	—
	Web	31.52 ± 3.94	−2.03 ± 1.68	**0.001**			
	Control	36.36 ± 4.87	−1.69 ± 1.41	**0.003**			
Muscle mass (kg)	F2F	44.29 ± 4.75	+ 0.16 ± 0.22^a^	**0.002**	8.462	**0.015**	F2F vs. C: *p* = 0.008; Web vs. C: *p* = 0.006
	Web	43.67 ± 4.37	+ 0.16 ± 0.18^a^	**0.001**			
	Control	44.20 ± 4.42	−0.23 ± 0.35	0.260			
Predicted VO₂max	F2F	29.19 ± 1.96	+ 0.87 ± 1.11	**0.006**	7.683	**0.021**	Web vs. C: *p* = 0.007
(ml·kg⁻¹·min⁻¹)	Web	29.30 ± 1.62	+ 0.65 ± 0.83^a^	**0.007**			
	Control	29.89 ± 1.45	**−0.03 ± 0.40**	0.705			

^a^Significantly greater change than control

Bold values indicate statistical significance (*p* < 0.05)

Health-related quality of life was evaluated across all eight SF-36 domains; change scores and between-group comparisons are presented in Table 6.

**Table 6:** SF-36 quality of life change scores (Δ) and between-group comparisons

SF-36 Domain	F2F Δ	Web Δ	Control Δ	*p* (Δ)	Post-hoc
Physical Functioning	+ 30.50 ± 16.61	+ 33.25 ± 19.95^a^	+ 16.00 ± 16.51	**0.016**	Web vs. C: *p* = 0.007
Role-Physical	+ 38.75 ± 38.07^a^	+ 32.50 ± 63.84	+ 2.50 ± 49.93	**0.043**	F2F vs. C: *p* = 0.012
Role-Emotional	+ 55.00 ± 57.69^a^	+ 45.00 ± 62.47	+ 3.33 ± 59.98	**0.032**	F2F vs. C: *p* = 0.010
Vitality	+ 7.25 ± 17.65	+ 10.25 ± 17.90^a^	−1.75 ± 19.99	**0.039**	Web vs. C: *p* = 0.011
Mental Health	−1.80 ± 5.86	−1.80 ± 6.49	−1.00 ± 9.12	0.979	—
Social Functioning	+ 11.88 ± 16.53	+ 19.38 ± 23.36^a^	+ 1.25 ± 25.59	**0.019**	Web vs. C: *p* = 0.008
Bodily Pain	+ 15.75 ± 20.97	+ 10.13 ± 20.37	+ 2.00 ± 29.62	0.083	—
General Health	+ 9.75 ± 11.13^a^	+ 3.75 ± 6.50^a^	−3.50 ± 14.51	**0.001**	F2F vs. C: *p* = 0.001; Web vs. C: *p* = 0.009

^a^Significantly greater change than control. Higher Δ = greater improvement

Bold values indicate statistical significance (*p* < 0.05)

To facilitate clinical interpretation and support future meta-analyses, Cohen’s d effect sizes for all pairwise between-group comparisons are summarised in Table 7.

**Table 7:** Cohen’s d effect sizes for between-group comparisons of change scores

Outcome	F2F vs. Control	Web vs. Control	F2F vs. Web
MRS Total	−1.16 (L)	−0.76 (M)	−0.42 (S)
SF-36 Physical Functioning	0.63 (M)	0.75 (M)	−0.13 (N)
SF-36 General Health	0.94 (L)	0.80 (M)	0.46 (S)
Beck Depression Inventory	−0.67 (M)	−0.92 (L)	0.28 (S)
Fatigue Severity Scale	−1.04 (L)	−1.66 (L)	0.20 (S)
Pittsburgh Sleep Quality Index	−1.08 (L)	−1.19 (L)	0.15 (N)
Body Fat (%)	0.09 (N)	−0.11 (N)	0.29 (S)
Muscle Mass (kg)	0.37 (S)	0.38 (S)	**0.00 (N)**
Predicted VO₂max	1.08 (L)	1.04 (L)	0.23 (S)

Negative values for MRS, BDI, FSS, PSQI indicate greater improvement in the first group. Positive values for SF-36 and VO₂max indicate greater improvement in the first group

*N *negligible*, S *small*, M *medium*, L *large

Bold values indicate statistical significance (*p* < 0.05)

No adverse events or exercise-related injuries were reported during the 8-week intervention period.

## Discussion

This three-arm randomised controlled trial found that an 8-week progressive programme of combined aerobic and resistance training improved menopausal symptoms, quality of life, psychological well-being, and predicted VO₂max in early postmenopausal women. Web-based delivery through PhysiApp performed similarly to face-to-face supervision across most outcomes, with between-group effect sizes remaining in the negligible-to-small range.

The MRS total score reductions in both exercise groups (Δ = −12.90 and − 9.95) exceeded those reported in comparable trials: Yılmaz Babacan et al. [33] reported MRS reductions of approximately 6–8 points following 12 weeks of combined training, and Berin et al. [55] reported moderate improvements following 15 weeks of resistance training alone. The face-to-face group showed a large effect relative to controls (d = − 1.16) and the web-based group a medium effect (d = − 0.76). A recent overview of systematic reviews by Money et al. [32] examined physical activity and exercise interventions for menopausal symptoms across multiple scales and found some evidence supporting exercise for symptom relief, but concluded that evidence was insufficient to recommend a specific modality — underscoring the contribution of the present trial.

The finding that web-based delivery matched face-to-face supervision is noteworthy. Despite the lack of direct contact, the app-based group reached similar endpoints in every domain, with most between-intervention effect sizes below 0.2. Tarakcı et al. [56] reported comparable findings in multiple sclerosis patients, where telerehabilitation and supervised exercise produced equivalent outcomes. However, we acknowledge that the absence of objective adherence data in our study limits the strength of this comparison. While Bennell et al. [41] demonstrated that web-based platforms can improve exercise adherence in musculoskeletal populations, our inability to retrieve app usage records and the absence of home exercise logs prevent us from drawing direct parallels regarding adherence. Future studies should integrate prospective adherence monitoring from the outset.

The psychological outcomes deserve comment. Effect sizes for fatigue reduction were among the largest in the study (d = − 1.04 to − 1.66), and sleep quality improvements were similarly strong (d = − 1.08 to − 1.19). Depression, fatigue, and poor sleep have been shown to cluster during the menopausal transition and reinforce one another [23, 24]. The reductions we observed across all three domains suggest that structured exercise may interrupt this cycle. Notably, SF-36 Mental Health scores did not improve in any group and showed a slight decline; however, this domain was significantly higher in the control group at baseline, suggesting a ceiling effect and possible regression to the mean rather than a true deterioration. Structured aerobic exercise has also been shown to improve health-related quality of life in other chronic populations, including coronary artery disease [57], further supporting the broad benefits of regular physical activity.

Muscle mass increased modestly in both exercise groups (d = 0.37–0.38 vs. control), consistent with evidence that various exercise modalities can positively influence musculoskeletal outcomes in postmenopausal women [58]. Recent experimental data by Gould et al. [13] and Baker et al. [15] demonstrate that body composition and metabolic parameters continue to change during the menopausal transition, supporting the rationale for exercise intervention during this window. Body fat percentage, by contrast, showed similar within-group reductions across all three groups, with negligible between-group effect sizes — a pattern that likely reflects the short intervention duration and the absence of a dietary component.

The mean improvement in predicted VO₂max was modest (0.65–0.87 ml·kg⁻¹·min⁻¹), and we acknowledge that changes of this magnitude may fall within the range of day-to-day measurement variability for submaximal testing. While the between-group effect sizes were large (d > 1.0), the absolute improvements should be interpreted cautiously given the limitations of submaximal estimation.

### Strengths and limitations

Strengths of this study include the three-arm design allowing direct comparison of delivery methods, comprehensive effect-size reporting to support future meta-analyses, validated instruments in Turkish populations, and the use of a commercially available telerehabilitation platform that can be reproduced in routine clinical settings.

Several limitations warrant detailed discussion. First, participants and the treating physiotherapist could not be blinded to group allocation, introducing potential performance and detection bias, particularly for self-reported outcomes. Future trials could employ attention-matched control conditions to mitigate this.

Second, objective adherence data were not available for either exercise group. Face-to-face participants attended all four supervised sessions, but session-by-session home exercise completion was not logged. For the web-based group, platform data retention policies prevented retrospective retrieval of usage records. Without these data, we cannot confirm that both groups completed equivalent exercise volumes, which limits the validity of the comparability claim. Future trials should integrate prospective digital monitoring and standardised exercise diaries from study inception.

Third, predicted VO₂max was estimated using a submaximal treadmill protocol rather than direct gas exchange measurement. Submaximal estimates have known limitations including day-to-day variability of ± 1–2 ml·kg⁻¹·min⁻¹ and reliance on assumptions about maximal heart rate that may not hold in menopausal women. Direct measurement by cardiopulmonary exercise testing would provide more accurate and clinically interpretable fitness data.

Fourth, body composition was assessed by BIA (TANITA BC-545 N) rather than DXA, the current gold standard. BIA may overestimate fat-free mass and underestimate fat percentage, and its sensitivity to small changes over short periods is limited. The BIA-derived variables we excluded from the revised manuscript (BMR, metabolic age, bone mass) are acknowledged to lack adequate accuracy for research purposes.

Fifth, all psychological and symptom outcomes relied on self-reported questionnaires, which are subject to recall bias, social desirability, and expectation effects — especially in an unblinded trial.

Sixth, the 8-week follow-up was too short to determine whether benefits persist, stabilise, or reverse after programme completion. Seventh, recruitment from a single geographic region in Istanbul may limit generalisability to other cultural and socioeconomic settings. Eighth, the trial was registered after enrolment commenced; although the protocol was not modified, delayed registration represents a methodological shortcoming. Finally, no cost-effectiveness data were collected, leaving the economic implications of each delivery model unknown.

## Conclusions

Eight weeks of combined aerobic and resistance training reduced menopausal symptom severity, improved multiple domains of health-related quality of life, reduced fatigue, and improved sleep quality in early postmenopausal women compared with an education-only control. Web-based exercise also significantly reduced depressive symptoms; the face-to-face group showed a similar trend that did not reach statistical significance after correction for multiple comparisons. Muscle mass increased modestly in both exercise groups; however, between-group differences in body fat percentage were negligible. Web-based delivery through a mobile application produced effects comparable to face-to-face supervision for most outcomes, though the absence of objective adherence data limits the strength of this comparison. App-based exercise may offer a practical alternative for women who cannot attend in-person sessions. Future trials should examine longer follow-up periods, larger and more diverse samples, integrated adherence monitoring, the combined effects of exercise with dietary counselling and/or hormone replacement therapy, and cost-effectiveness of different delivery models, ideally with gold-standard body composition measures such as DXA.

## Data Availability

Available from the corresponding author on reasonable request.
